# Treatment with Hydrogen-Rich Water Improves the Nociceptive and Anxio-Depressive-like Behaviors Associated with Chronic Inflammatory Pain in Mice

**DOI:** 10.3390/antiox11112153

**Published:** 2022-10-30

**Authors:** Santiago Coral-Pérez, Ignacio Martínez-Martel, Maria Martínez-Serrat, Gerard Batallé, Xue Bai, Christie R. A. Leite-Panissi, Olga Pol

**Affiliations:** 1 Grup de Neurofarmacologia Molecular, Institut d’Investigació Biomèdica Sant Pau (IIB SANT PAU), Sant Quintí 77-79, 08041 Barcelona, Spain; 2 Grup de Neurofarmacologia Molecular, Institut de Neurociències, Universitat Autònoma de Barcelona, 08193 Barcelona, Spain; 3 Department of Psychology, Faculty of Philosophy Science and Letters of Ribeirão Preto, University of São Paulo, Ribeirão Preto 14040-901, SP, Brazil

**Keywords:** allodynia, anxiety, apoptosis, depression, hyperalgesia, inflammatory pain, molecular hydrogen, oxidative stress

## Abstract

Chronic inflammatory pain is manifested in many diseases. The potential use of molecular hydrogen (H_2_) as a new therapy for neurological disorders has been demonstrated. Recent studies prove its analgesic properties in animals with neuropathic pain, but the possible antinociceptive, antidepressant, and/or anxiolytic actions of H_2_ during persistent inflammatory pain have not been investigated. Therefore, using male mice with chronic inflammatory pain incited by the subplantar injection of complete Freud’s adjuvant (CFA), we assessed the actions of hydrogen-rich water (HRW) systemically administered on: (1) the nociceptive responses and affective disorders associated and (2) the oxidative (4-hydroxy-2-nonenal; 4-HNE), inflammatory (phosphorylated-NF-kB inhibitor alpha; p-IKBα), and apoptotic (Bcl-2-like protein 4; BAX) changes provoked by CFA in the paws and amygdala. The role of the antioxidant system in the analgesia induced by HRW systemically and locally administered was also determined. Our results revealed that the intraperitoneal administration of HRW, besides reducing inflammatory pain, also inhibited the depressive- and anxiolytic-like behaviors associated and the over expression of 4-HNE, p-IKBα, and BAX in paws and amygdala. The contribution of the nuclear factor erythroid 2-related factor 2/heme oxygenase 1 and NAD(P)H: quinone oxidoreductase 1 pathway in the analgesic activities of HRW, systemically or locally administered, was also shown. These data revealed the analgesic, antidepressant, and anxiolytic actions of HRW. The protective, anti-inflammatory, and antioxidant qualities of this treatment during inflammatory pain were also demonstrated. Therefore, this study proposes the usage of HRW as a potential therapy for chronic inflammatory pain and linked comorbidities.

## 1. Introduction

Chronic pain is generally defined as a syndrome characterized by persistent suffering that is prolonged for long periods and is challenging to alleviate/eradicate in clinical practice [1]. Different comorbidities commonly accompany chronic pain, for example, cognitive deficits, sleep difficulties, and affectations on the emotional mood, especially depressive and anxiety disorders [2,3].

In several preclinical models of chronic pain, such as induced by the complete or partial constriction of the sciatic nerve in the case of neuropathic pain [4,5,6,7], a close relationship between chronic pain and alterations in the affective states related with anxiety- and depressive-like behaviors has been found. In contrast, a possible relationship between chronic pain generated by peripheral inflammation and the concurrent development of anxiety- and depressive-like behaviors has not been fully established. While some authors showed that chronic inflammatory pain is accompanied by depressive-like behaviors [3,8,9], others only estimated the anxiety-like behaviors associated with inflammatory pain [2,10,11,12]. Thus, one of our objectives is to examine the plausible development of both affective deficits during chronic inflammatory pain generated by the subplantar injection of complete Freund’s adjuvant (CFA) in mice.

Several studies demonstrated the participation of pro-inflammatory cytokines, for instance interleukins (IL), IL-1α, IL-1β, TNFα, and IL-6, in the progress of inflammatory pain [13,14]. Oxidative stress and the nuclear factor-κB (NF-κB) activated by the nuclear factor of kappa light polypeptide gene enhancer in the B-cells inhibitor, alpha (IKBα), are two notable factors that significantly contribute to the progress of inflammatory pain through regulating a large collection of genes involved in different processes of the inflammatory replies [15,16,17]. Several studies further revealed the apoptotic reactions produced by inflammation in the peripheral and central nervous system (CNS) of animals with chronic inflammatory pain [18].

A solid relationship between oxidative stress and inflammation with depressive and/or anxiety disorders has been revealed; indeed, an increased expression of oxidative and inflammatory biomarkers in specific brain areas of depressive patients was demonstrated [19,20]. Consequently, a therapy for mood disorders which focuses on the use of antioxidants as an antidepressant and/or anxiolytic agents, avoiding the important side effects induced by classic antidepressants and/or anxiolytics, was proposed [20]. In accordance, the administration of nuclear factor erythroid 2-related factor 2 (Nrf2) transcription factor activators, such as sulforaphane or oltipraz, inhibited the depressive- and anxiety-like behaviors accompanying chronic neuropathic pain in mice [21,22], and TBE-31 and MCE-1 are two possible candidates for treating the inflammation-associated depressive behaviors [23].

In the last years, some researchers demonstrated the beneficial effects of molecular hydrogen (H_2_). Currently, numerous studies propose the use of H_2_ as a therapy, because of: a) its facility to cross the blood–brain barrier and penetrate cell membranes, b) the lack of evidence revealing undesired side effects or the development of tolerance induced by the repetitive treatment with H_2_, and c) the antioxidant actions of this molecule against various oxidative-related diseases [24,25]. The antioxidant effects of H_2_ are predominantly adjudicated to its capacity to incite the translocation of the Nrf2 transcription factor from the cytosol to the nucleus, with the following transcription of several genes, such as superoxide dismutase (SOD), heme oxygenase 1 (HO-1), and NAD(P) H:quinone oxidoreductase 1 (NQO1) [25,26].

Other studies have also shown the anti-inflammatory responses induced by H_2_ through inhibiting pro-inflammatory cytokines, for instance, interleukins IL-1β, IL-4, IL-5, and IL-13, among others [27,28]. In the last years, the potential antidepressant and/or anxiolytic effects of H_2_ have also been shown in animals with unpredictable mild stress [29] and in healthy patients [30]. All these attributes suggested that H_2_ might be a good candidate for treating chronic pain and the linked affective disorders. Consequently, several authors have revealed the painkiller properties of this gas in animals with neuropathic pain [31,32]. Even so, its possible antinociceptive, antidepressant, and/or anxiolytic effects during chronic inflammatory pain have not been completely established.

In mice with chronic inflammatory pain provoked by CFA, our aims are to evaluate: (1) the antinociceptive effects of the intraperitoneal and/or subplantar administration of hydrogen-rich water (HRW), (2) the feasible antidepressant and/or anxiolytic effects of HRW during chronic inflammatory pain, (3) the main pathways that take part in the antinociceptive actions of HRW, and (4) the effects of HRW in the oxidative, inflammatory, and/or apoptotic reactions generated by CFA in the paws and amygdala, as key structures in regulating the nociceptive [33] and emotional responses [2].

## 2. Materials and Method

### 2.1. Animals

The experimentations were achieved with 6–8 week old male C57BL/6 mice (25–26 g) purchased at Envigo Laboratories (Barcelona, Spain), maintained under 12/12 h light/dark conditions in a space acclimatized to 22 °C and a humidity of 66% until use. All experiments were carried out with the guidelines of the European Commission’s directive (2010/63/EC) and the Spanish Law (RD 53/2013) that regulated animal research and were authorized by the local Committee of Animal Use and Care of the Autonomous University of Barcelona (ethical code: 9863). All endeavors were performed to diminish the suffering and the number of animals used.

### 2.2. Generation of Inflammatory Pain

To induce inflammatory pain, 30 μL of CFA (Sigma-Aldrich, St. Louis, MO, USA) was injected into the right hind paw via the subplantar method. For this process, animals were anesthetized with isoflurane (3% induction, 2% maintenance), applying the protocol used in a previous study [34]. Control animals were treated with the equal amount of saline (NaCl 0.9%; SS).

### 2.3. Mechanical Allodynia

Mechanical allodynia was estimated by determining the hind limb withdrawal responses after its stimulation with the von Frey filaments, for which the flexion forces were between 0.4 g to 3.0 g. Animals were sited in Plexiglas cylinders (25 cm × 10 cm) on a platform of a wire grid where the von Frey filaments (North Coast Medical, Inc., San Jose, CA, USA) were applied. The test began with the 0.4 g filament, and the strength of the next filaments enhanced or diminished depending on whether the animal removed, licked, or shook the paw or not. The hind limb withdrawal response was evaluated by applying an Excel program (Microsoft Iberia SRL, Barcelona, Spain), using the data sequence of five applications of filaments.

### 2.4. Thermal Hyperalgesia

Thermal hyperalgesia was assessed by measuring the hind limb withdrawal latency response in seconds when the animal was exposed to heat in the plantar test (Ugo Basile, Varese, Italy). Animals were put in Plexiglas cylinders (25 cm × 10 cm) over a crystal superficies. The heat supply was placed below the paw of the animal and activated to light up until the animal reacted, or up to 12 s. Mean paw withdrawal latencies were taken from three separate measurements of hind limb withdrawal. 

In all tests, both hind paws were evaluated. Animals were familiarized to the von Frey and plantar environment for 1 h prior to the test to permit correct immobility behavior.

### 2.5. Depressive-like Behaviors

The assessment of the depressive-like behaviors was accomplished at day 16 after CFA injection by using the tail suspension test (TST) and forced swimming test (FST), where the immobility of the animals was calculated in seconds.

In the TST, animals were individually suspended by the tail from a platform, sited at 35 cm high from the ground, utilizing adhesive tape (1 cm). Animals were recorded by a digital camera and the immobility time was quantified over 6 min [5,35].

Concerning the FST, each animal was put in a Plexiglas tube (25 cm × 10 cm) that contained water to 10 cm of deepness at 24 °C. Mice were subjected to forced swimming for 6 min, and the duration of immobility was quantified during the last 4 min, when mice showed an adequately stable level of immobility, in accordance with [36,37,38].

In both tests, mice were familiarized to the testing space for 1 h before starting the test.

### 2.6. Anxiety-like Behaviors

The anxiety-like behaviors were tested by using the elevated plus maze (EPM) as it was described by Kraeuter et al. (2019) [39] and open field (OF) by Kraeuter et al. (2019) [40]. The EPM is an apparatus with four arms orientated on a cross shape, each one of 5 cm wide and 35 cm long, where two of the arms stay open and the other two are closed, with walls of 15 cm. The distance of the EPM to the ground is 45 cm. Mice were sited in the center of the maze facing one of the open arms, and their behavior was filmed for 5 min. The number of entries in the open and closed arms and the percentage of time spent in the open arms were calculated for each animal.

In the OF test, mice were positioned in the arena of a box (44 cm × 44 cm) with a grey non-reflecting base and four walls (30 cm high), and the behavior was recorded for 5 min. Animals were permitted to move freely across the apparatus and explore the environment. The number of entries and percentage of time passed in the central area and the number of squares crossed were quantified.

In both tests, animals were familiarized to the testing space for 1 h before starting the evaluation.

### 2.7. Experimental Procedures

Since one of our goals was to examine the possible development of both affective deficits (depressive and anxious behaviors) associated with chronic inflammatory pain generated by the subplantar injection of CFA, the evaluation of mechanical allodynia and thermal hyperalgesia was performed between days 13 and 16 after CFA injection, and affective responses were assessed on day 16 after CFA injection.

In the first procedure, SS- or CFA-injected mice were intraperitoneally administered with vehicle (VEH) or HRW (0.3 mM) at one time/day (HRW 1T) or two times/day (HRW 2T) over three consecutive days. Allodynia and hyperalgesia were tested prior to CFA injection (day 0), one day before treatment (day 13 after CFA injection) and in each day of treatment (days 14–16 after CFA-injection) (*n* = 6 animals for group).

In separate groups of SS- or CFA-injected mice, the antiallodynic and anti-hyperalgesic effects induced by the subplantar administration of HRW (0.3 mM) or VEH, injected at 2T per day from days 14 to 16 after CFA injection, were also evaluated (*n* = 6 animals for group).

In distinct groups of animals, the evaluation of depressive- and anxiety-like behaviors was performed at day 16 after CFA injection. The possible antidepressant and/or anxiolytic effects produced by the 2T daily intraperitoneal administration of HRW from days 14 to 16 after CFA injection were also evaluated in the TST/FST and EPM/OF tests, respectively (*n* = 8 animals for each group). Animals treated with VEH were used as controls.

Animals were tested 1 h after HRW or VEH injection [41].

To examine the probable involvement of the Nrf2/HO-1 and NQO1 pathway in the analgesic actions of HRW, the effects of the intraperitoneal and subplantar administration of HRW or VEH, injected at 2T or 1T per day, in animals intraperitoneally or subplantarly co-treated with 25 mg/kg or 625 μg of ML385, a Nrf2 inhibitor [42], 10 mg/kg or 250 μg of tin protoporphyrin IX (SnPP), an HO-1 inhibitor [43], 10 mg/kg or 250 µg of dicoumarol, a NQO1 inhibitor [44], or VEH (DMSO 1% in SS) were assessed (*n* = 6 animals for group). The HRW concentration and doses of ML-385, SnPP and dicoumarol were chosen in line with earlier studies [41,45].

At 16 days after CFA injection, animals injected with HRW or VEH were euthanized by cervical dislocation, and the expression of 4-hydroxy-2-nonenal (4-HNE), Bcl-2-like protein 4 (BAX), and phosphorylated IKBα (p-IKBα) were assessed in the paws and amygdala tissues using Western blot.

All experiments were performed by a researcher blinded to the experimental conditions.

### 2.8. Drugs

HRW was prepared using a hydrogen water generator from Hydrogen (Osmostar Soriano S.L., Alicante, Spain), which uses the electrolysis method to break down H_2_O into H_2_. SnPP was obtained from Frontier Scientific (Livchem GmbH & Co., Frankfurt, Germany) and ML-385 and dicoumarol from Eurodiagnostico S.L (Madrid, Spain); all were dissolved in DMSO 1% in SS.

All drugs were intraperitoneally and subplantarly injected in a volume of 10 mL/kg and 30 μL, respectively. HRW was administered 1 h before testing, while ML-385, SnPP, and dicoumarol were administered at 30 min before the tests, in accordance with earlier studies [41,45]. All drugs were newly prepared prior to their administration. For each group treated with a drug, the corresponding control group received the identical volume of corresponding VEH.

### 2.9. Western Blot Analysis

Sixteen days following CFA or SS injection, animals were euthanized by cervical dislocation, and the ipsilateral subplantar tissue of the hind paws and contralateral amygdala were removed, frozen, and kept at −80 °C until usage. We evaluated the levels of 4-HNE, p-IKBα, and BAX. Tissues were sonicated (amygdala) or homogenized (paw) in cold lysis buffer RIPA Buffer (Sigma-Aldrich, St. Louis, MO, USA). Next to the solubilisation for 1 h at 4 °C, tissues were sonicated (10 s) and centrifuged at 700× *g* at 4 °C (20 min). The supernatant (60 μg of total protein) mixed with Laemmli loading buffer was loaded onto 4% stacking/12% separating sodium dodecyl sulfate-polyacrylamide gels. Afterward, proteins were electrophoretically transferred on a polyvinylidene fluoride membrane for 120 min and blocked with Tris-buffered saline with Tween 20 and nonfat dry milk, bovine serum albumin (5%), or phosphate-buffered saline with Tween 20 and nonfat dry milk (5%) for 75 min. Then, membranes were incubated overnight at 4 °C with specific primary rabbit antibodies against 4-HNE (1:100; Abcam, Cambridge, UK), p-IKBα (1:150; Abcam, Cambridge, UK), IKBα (1:100; Abcam, Cambridge, UK), BAX (1:150; Cell Signaling Technology, Danvers, MA, USA), and GAPDH (1:5000; Merck, Billerica, MA, USA). After that, blots were incubated at room temperature (60 min) with a horseradish peroxidase-conjugated anti-rabbit secondary antibody (GE Healthcare, Little Chalfont, UK), proteins were detected by using chemiluminescence reagents (ECL kit; GE Healthcare, Little Chalfont, Buckinghamshire, UK), and band density was evaluated by densitometry with the Image-J program (National Institutes of Health, Bethesda, MD, USA).

### 2.10. Statistical Analysis

All results were represented as the mean values ± standard error of the mean (SEM). To evaluate the effects of inflammation, treatment, and time, and the interactions between them, the three-way analysis of variance (ANOVA) with repeated measures was performed using the Statistical Package for Social Sciences (SPSS, Version 17.0, IL, USA). For each day, the effects produced by different therapies in the allodynia, hyperalgesia, and anxiety–depressive-like behaviors were analyzed with a one-way ANOVA and, subsequently, a Student–Newman–Keuls test using the Graph Pad Prism program (version 8 for Windows). A value of *p* < 0.05 was considered significant.

## 3. Results

### 3.1. Effects of the Administration of HRW in the Mechanical Allodynia and Thermal Hyperalgesia Caused by CFA

Initially, we assessed the effects of HRW injected intraperitoneally at 1T or 2T per day for 3 consecutive days on the allodynia and hyperalgesia caused by peripheral inflammation on days 14 to 16 after CFA injection. In both tests, the three-way repeated measures ANOVA displayed significant effects of inflammation, treatment, and time (*p* < 0.001) and interactions among inflammation × treatment, inflammation × time, treatment × time, as well as between inflammation × treatment × time (*p* < 0.001).

**Figure 1:**
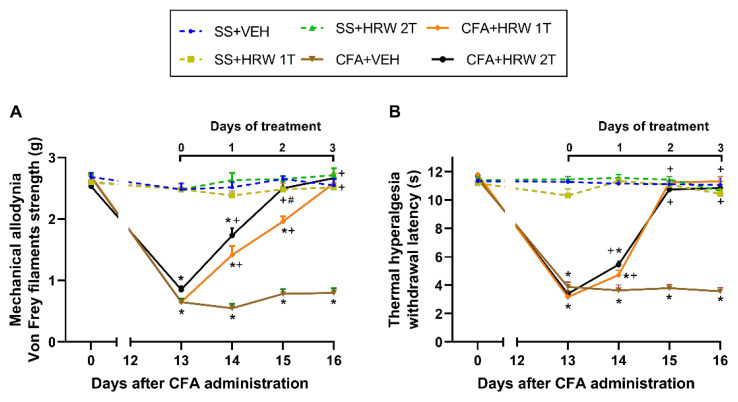
Effects of the intraperitoneal injection of HRW or VEH on the mechanical allodynia and thermal hyperalgesia induced by paw inflammation. Effects of the repetitive administration of HRW or VEH, injected at 1T and 2T per day, over the decreased von Frey filaments strength (g) (**A**) and the withdrawal latency (s) (**B**) generated by CFA in the ipsilateral hind paws. For each test, * indicates significant changes vs. their respective SS-injected mice (SS + VEH, SS + HRW 1T, or SS + HRW 2T), + represents significant differences vs. CFA + VEH injected mice, and # denotes significant differences vs. CFA + HRW 1T (*p* < 0.05, on one-way ANOVA and Student–Newman–Keuls test). Data are presented as mean values ± SEM; *n* = 6 animals for group.

Thus, the reduced threshold of the ipsilateral hind paw withdrawal to von Frey filaments stimulus provoked by CFA at 13 days after its injection (*p* < 0.001, one-way ANOVA vs. SS-injected mice injected with VEH; Figure 1A) was fully inhibited after 2 and 3 days of treatment with HRW injected at 2T or 1T per day, respectively. Moreover, the antiallodynic effects induced by the double administration of HRW at two days of treatment were higher than those produced by the single administration (*p* < 0.001, one-way ANOVA).

Our data further showed that the reduced withdrawal thresholds of the ipsilateral hind paws of CFA-injected animals in reaction to a thermal stimulus, at 13 days after its injection (*p* < 0.001, one-way ANOVA vs. SS-injected mice treated with VEH; Figure 1B), were entirely blocked at 2 days of treatment with HRW injected a 1T or 2T per day.

In all tests and for each time evaluated, no significant differences were observed between SS-injected mice treated with VEH or HRW at 1T or 2T per day (Figure 1). In both tests, the intraperitoneal administration of HRW or VEH at 1T or 2T per day did not produce any effects in the contralateral paws of CFA- or SS-injected mice (data not displayed).

**Figure 2:**
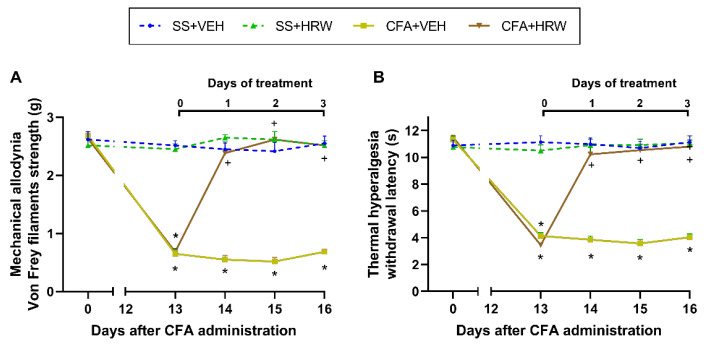
Effects of the subplantar administration of HRW or VEH on the mechanical allodynia and thermal hyperalgesia generated by paw inflammation. Effects of the subplantar administration of HRW or VEH, injected at 2T per day, over the decreased von Frey filaments strength (g) (**A**) and withdrawal latency (s) (**B**) provoked by CFA in the ipsilateral hind paws. For each test, * indicates significant variations vs. SS-injected mice treated with VEH (SS + VEH), and + represents significant differences vs. CFA-injected mice treated with VEH (CFA + VEH) (*p* < 0.05, one-way ANOVA and Student–Newman–Keuls test). Data are shown as mean values ± SEM; *n* = 6 animals for group.

The mechanical antiallodynic and thermal antihyperalgesic effects produced by the subplantar injection of HRW, administered at 2T per day, in mice with inflammatory pain were also evaluated (Figure 2). The three-way repeated measures ANOVA displayed significant effects of inflammation, treatment, and time (*p* < 0.001) and interactions between inflammation × treatment, inflammation × time, treatment × time, as well as between inflammation × treatment × time (*p* < 0.001) for both the mechanical allodynia and thermal hyperalgesia.

Results showed a total reversion of CFA-induced allodynia (Figure 2A) and hyperalgesia (Figure 2B) on day 1 of treatment with HRW. In concordance, the antiallodynic and antihyperalgesic effects generated by subplantar administration of HRW were greater than those made by VEH (*p* < 0.001; one-way ANOVA and Student–Newman–Keuls test).

In both tests, no significant differences were detected in the ipsilateral paws of SS-injected mice subplantarly administered with HRW or VEH (Figure 2). In both paradigms, the local administration of HRW or VEH did not have any effects in the contralateral paws of CFA- or SS-injected mice (data not shown).

### 3.2. Inhibition of the Depressive-like Behaviors Associated with Inflammatory Pain Produced by the Intraperitoneal Administration of HRW

**Figure 3:**
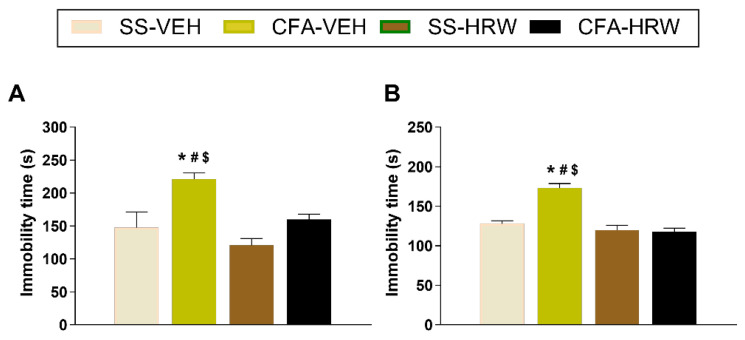
Treatment with HRW inhibited the depressive-like behaviors linked with chronic inflammatory pain. Immobility times (s) in the TST (**A**) and FST (**B**) at day 16 after CFA injection in animals intraperitoneally treated with HRW or VEH, injected at 2T per day for two consecutive days, are represented. The effects of HRW and VEH in SS-injected mice are also shown. For each test evaluated, * denotes significant differences vs. SS-injected mice treated with VEH, # vs. SS-injected mice treated with HRW, and $ vs. CFA-injected mice treated with HRW (*p* < 0.05, one-way ANOVA and Student–Newman–Keuls test). Data are shown as mean values ± SEM; *n* = 8 animals for group.

Firstly, we analyzed the possible depressive-like behaviors associated with chronic inflammatory pain by using the TST and FST. A significant increase in the immobility time was observed in CFA-injected mice as compared with SS-injected mice in the TST (Figure 3A) and FST (Figure 3B) (*p* < 0.0001; one-way ANOVA and Student–Newman–Keuls test), thus revealing that chronic inflammatory pain in mice is accompanied with depressive-like behaviors. We also evaluated whether the intraperitoneal administration of HRW could normalize this mood disorder. Our results showed that treatment with HRW intraperitoneally administered at 2T per day over two consecutive days normalized the increased immobility time observed in CFA-injected animals treated with VEH in the TST (*p* < 0.0001; one-way ANOVA and Student–Newman–Keuls test) and FST (*p* < 0.0001; one-way ANOVA and Student–Newman–Keuls test). This, therefore, suggests the antidepressant properties of HRW treatment in animals with inflammatory pain.

### 3.3. Inhibition of the Anxiety-like Behaviors Associated with Inflammatory Pain Produced by the Intraperitoneal Administration of HRW

**Figure 4:**
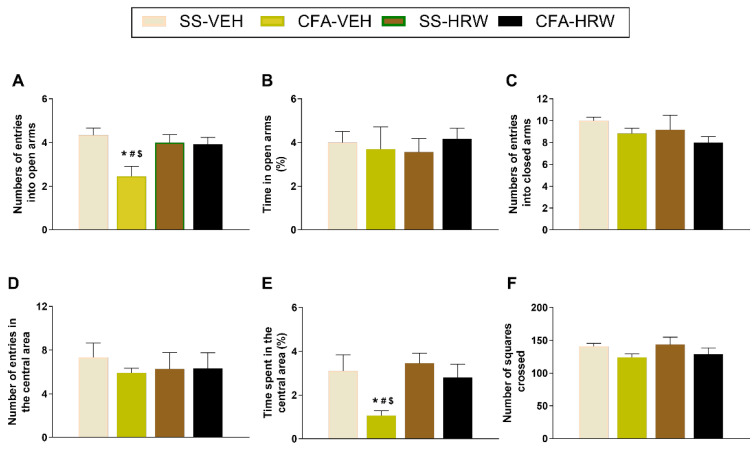
Treatment with HRW inhibited the anxiety-like behaviors linked with chronic inflammatory pain. The effects of the intraperitoneal treatment with HRW or VEH in the EPM and OF tests, at 16 days after CFA injection, are represented. In the EPM test, the number of entries to the open arms (**A**), the percentage of time spent in the open arms (**B**), and the number of entries into the closed arms (**C**) are shown. In the OF test, the number of entries in the central area (**D**), the percentage of time spent in the central area (**E**), and the number of squares crossed (**F**) are represented. The effects of HRW and VEH in SS-injected mice are also shown. For each test evaluated, * denotes significant differences vs. SS-injected mice treated with VEH, # vs. SS-injected mice treated with HRW, and $ vs. CFA-injected mice treated with HRW (*p* < 0.05; one-way ANOVA and Student–Newman–Keuls test). Data are presented as mean values ± SEM; *n* = 8 animals for group.

To study the possible anxiogenic-like behaviors associated with chronic inflammatory pain in mice, we analyzed their behavioral responses in the EPM and OF tests. Results revealed a significant decrease in the amount of entrances into the open arms in the EPM (*p* < 0.001; one-way ANOVA and Student–Newman–Keuls test; Figure 4A) and a reduced time spent in the central area of the OF test (*p* < 0.001; one-way ANOVA and Student–Newman–Keuls test; Figure 4E) of CFA-injected mice treated with VEH as compared with SS-injected animals treated with VEH. Thus, the anxiety-like behaviors accompanying chronic inflammatory pain are shown. In addition, our results demonstrated the normalization of these responses produced by the intraperitoneal administration of HRW in the EPM and OF tests, thus indicating the anxiolytic effects of this treatment during peripheral inflammation. No changes in the percent of time spent in the open arms (Figure 4B) or in the number of entries in the closed arms (Figure 4C) in the EPM, and neither in the number of entries into the central area (Figure 4D) nor in the number of squares crossed in the OF test (Figure 4F), were found between CFA- or SS-injected animals treated with VEH or HRW.

### 3.4. Reversion of the Antiallodynic and Antihyperalgesic Effects of HRW with Their Co-Treatment with Specific Inhibitors of the Nrf2/HO-1-NQO1 Signaling

**Figure 5:**
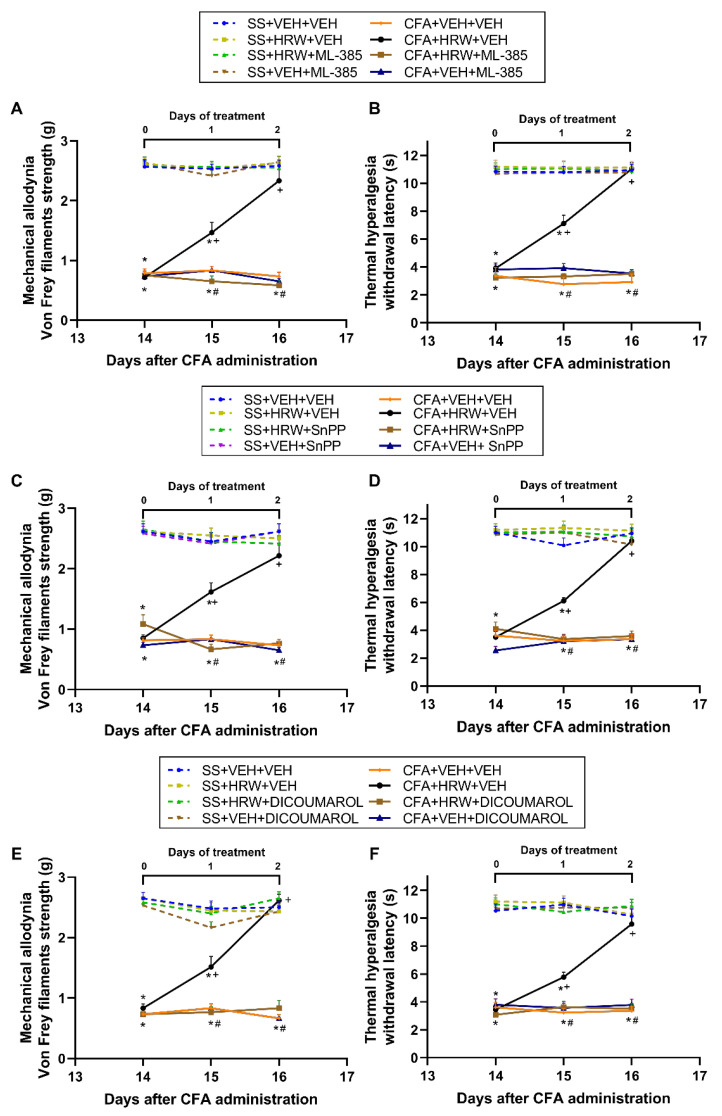
Reversion of the analgesic effects of HRW produced by inhibitors of the Nrf2/HO-1-NQO1 signaling in animals with inflammatory pain. Effects of the intraperitoneal co-administration of HRW with a Nrf2 inhibitor (ML-385, 25 mg/kg) (**A**,**B**), an HO-1 inhibitor (SnPP, 10 mg/kg) (**C**,**D**), a NQO1 inhibitor (dicoumarol, 10 mg/kg) (**E**,**F**), or VEH in the mechanical allodynia (**A**,**C**,**E**) and thermal hyperalgesia (**B**,**D**,**F**) provoked by CFA in the ipsilateral paws of mice are shown. For each test, * denotes significant differences vs. their respective SS-injected mice, + denotes significant differences vs. CFA-injected mice treated with VEH plus VEH, and # denotes significant differences vs. CFA-injected mice treated with HRW plus VEH (*p* < 0.05; one-way ANOVA and Student–Newman–Keuls test). Data are presented as mean values ± SEM; *n* = 6 animals for group.

The plausible contribution of the Nrf2/HO-1-NQO1 pathway in the pain-relieving actions induced by the intraperitoneal administration of HRW was studied by evaluating its effects in mice intraperitoneally co-administered with selective Nrf2 (ML-385, 25 mg/kg), HO-1 (SnPP, 10 mg/kg), or NQO1 (dicoumarol, 10 mg/kg) inhibitors on the allodynia and hyperalgesia instigated by paw inflammation (Figure 5).

For each co-treatment and test evaluated, significant effects of inflammation, treatment, time (*p* < 0.001), and interactions among them (*p* < 0.001) were demonstrated with the three-way repeated measures ANOVA.

Our findings showed the reversion of the antiallodynic (Figure 5A,C,E) and antihyperalgesic actions (Figure 5B,D,F) produced by the intraperitoneal administration of HRW in the ipsilateral paws of mice with inflammatory pain co-treated with ML-385 (Figure 5A,B), SnPP (Figure 5C,D), or dicoumarol (Figure 5E,F) after one and two days of treatment (*p* < 0.001; one-way ANOVA and Student–Newman–Keuls test vs. CFA-injected mice treated with HRW plus VEH).

Additionally, HRW intraperitoneally administered alone and combined with ML-385, SnPP, or dicoumarol did not have any effect in the ipsilateral paws of SS-injected animals (Figure 5) nor in the contralateral paws of SS- or CFA-injected mice (data not shown).

We also studied the role of the local Nrf2/HO-1-NQO1 path in the analgesic effects induced by the subplantar administration of HRW by evaluating the antinociceptive actions produced by the subplantar co-treatment of HRW with ML-385 (625 μg), SnPP (250 μg), or dicoumarol (250 μg).

**Figure 6:**
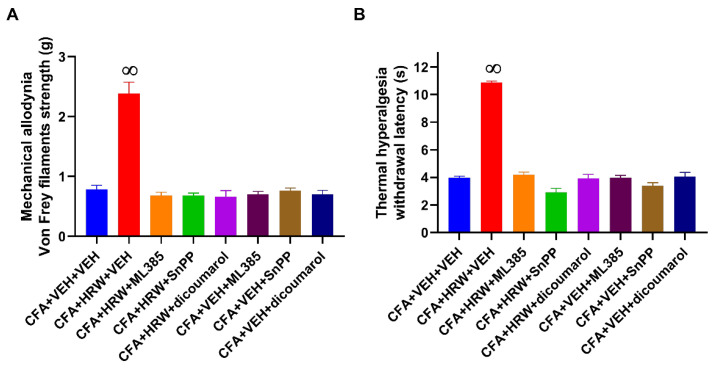
Reversion of the local analgesic actions of HRW produced by the Nrf2/HO-1-NQO1 signaling inhibitors during inflammatory pain. Effects of the subplantar co-administration of HRW with specific inhibitors of Nrf2 (ML-385, 650 μg), HO-1 (SnPP, 250 μg), and NQO1 (dicoumarol, 250 μg) or VEH on the mechanical allodynia (**A**) and thermal hyperalgesia (**B**) caused by CFA in the ipsilateral paws of mice are shown. For each test, ∞ denotes significant differences vs. other groups (*p* < 0.05; one-way ANOVA and Student–Newman–Keuls test). Data are presented as mean values ± SEM; *n* = 6 animals for group.

Our outcomes demonstrated that the antiallodynic (Figure 6A) and antihyperalgesic (Figure 6B) effects induced by the local administration of HRW were entirely reversed with the subplantar co-administration of ML-385, SnPP, or dicoumarol (*p* < 0.0001; one-way ANOVA and Student–Newman–Keuls test).

In addition, the subplantar administration of ML-385, SnPP, or dicoumarol alone did not modify the mechanical allodynia (Figure 6A) or thermal hyperalgesia (Figure 6B) caused by CFA. The subplantar administration of HRW alone and combined with ML-385, SnPP, or dicoumarol did not have any effect in the ipsilateral paw of SS-injected animals nor in the contralateral paws of SS- or CFA-injected mice (results not shown).

### 3.5. Effects of Treatment with HRW on the Expression of 4-HNE, BAX, and p-IKBα in the Paws of Animals with Peripheral Inflammation

**Figure 7:**
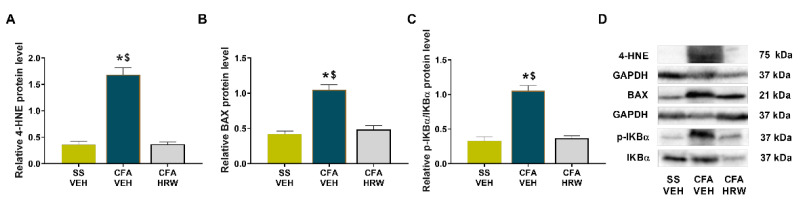
Effects of HRW on the expression of 4-HNE, BAX, and p-IKBα in the paw tissues of CFA-injected mice. The protein levels of 4-HNE (**A**), BAX (**B**), and p-IKBα (**C**) in the ipsilateral paws of CFA-injected mice treated with VEH or HRW are shown. SS-injected mice treated with VEH, used as controls, are also shown. Examples of blots for 4-HNE, BAX, and p-IKBα are displayed (**D**). 4-HNE and BAX are expressed relative to GAPDH levels, while p-IKBα is expressed relative to IKBα. In all graphics, * denotes significant differences vs. SS-injected mice plus VEH, and $ denotes significant differences vs. CFA-injected animals plus HRW (*p* < 0.05; one-way ANOVA and Student–Newman–Keuls test). Data are shown as the mean ± SEM; *n* = 3 samples.

We evaluated the effects of the intraperitoneal administration of HRW on the oxidative (4-HNE), apoptotic (BAX), and inflammatory reactions (p-IKBα) incited by CFA in paw tissues. Our results showed that CFA increased the expression of 4-HNE (Figure 7A), BAX (Figure 7B), and p-IKBα (Figure 7C) in the paws (*p* < 0.05; one-way ANOVA and Student–Newman–Keuls test compared with their respective SS plus VEH treated mice). Treatment with HRW normalized the overexpression of 4-HNE, BAX, and p-IKBα in the paw of mice with inflammatory pain.

### 3.6. Effects of Treatment with HRW on the Expression of 4-HNE, BAX, and p-IKBα in the Amygdala of Animals with Paw Inflammation

**Figure 8:**
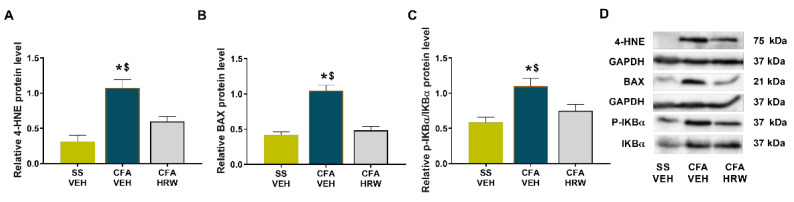
Effects of treatment with HRW on the expression of 4-HNE, BAX, and p-IKBα in the amygdala of CFA-injected mice. The protein levels of 4-HNE (**A**), BAX (**B**), and p-IKBα (**C**) in the amygdala of CFA-injected mice treated with VEH or HRW are shown. SS-injected mice treated with VEH, used as controls, are also shown. Examples of blots for 4-HNE, BAX, and p-IKBα are displayed (**D**). 4-HNE and BAX are expressed relative to GAPDH levels, while p-IKBα is expressed relative to IKBα. In all graphics, * denotes significant differences vs. SS-injected mice plus VEH, and $ denotes significant differences vs. CFA-injected animals plus HRW (*p* < 0.05; one-way ANOVA and Student–Newman–Keuls test). Data are shown as the mean ± SEM; *n* = 3 samples.

As occurs in the periphery, the increased protein levels of 4-HNE (Figure 8A), BAX (Figure 8B), and p-IKBα (Figure 8C) observed in the amygdala of animals with peripheral inflammation (*p* < 0.05; one-way ANOVA and Student–Newman–Keuls test as related with SS-injected animals treated with VEH) were reversed by HRW treatment.

## 4. Discussion

The present study revealed the antiallodynic and antihyperalgesic effects of HRW and its antidepressant and anxiolytic actions during inflammatory pain in mice. Data further showed the participation of the central and peripheral Nrf2/HO-1-NQO1 pathway in the analgesic actions of HRW and the modulator actions of this treatment in the oxidative, inflammatory, and apoptotic replies incited by CFA in the paws and amygdala. Treatment with H_2_ ameliorates different neurodegenerative disorders [46] and exerts powerful antioxidant, anti-inflammatory, and anti-apoptotic effects in several pathological conditions [24,47]. In addition, the antinociceptive properties of HRW in chronic pain have been previously found in animals with nerve injury-induced neuropathic pain [31,32]. Here, we demonstrated, for the first time, that the intraperitoneal and subplantar administration of HRW both inhibit the allodynia and hyperalgesia provoked by CFA. That is, the systemic treatment with HRW, administered at 1T or 2T per day, completely abolished the hyperalgesia after two days of treatment as well as the allodynic responses after three and two days of treatment, respectively, therefore, revealing the greater effectiveness of the double vs. the simple intraperitoneal injection per day of HRW in treating the allodynia. Interestingly, the subplantar injection of HRW inhibited both nociceptive responses (allodynia and hyperalgesia) with one day of treatment, displaying the major effectiveness of the local against the systemic treatment with HRW in modulating chronic inflammatory pain.

The link between chronic pain and multiple associated mood disorders has been described in many studies, principally in those performed in animal models of neuropathic pain [4,5,6,21,48,49]. In contrast, the appearance of more than one affective disorder linked with peripheral inflammatory pain has not been completely determined. In this study, performed at 16 days after CFA injection, the development of depressive- and anxiety-like behaviors in animals with inflammatory pain has been revealed. Our results supported the depressive-like behaviors [3,8,9] and the anxiety-like behaviors linked with inflammatory pain [2,10,11,12] and further demonstrated that both affective disorders are also manifested at the same time in mice with inflammatory pain, as occurs in neuropathic pain induced by nerve injury [5] or chemotherapy [50]. These findings show the importance of investigating for new options to reduce not only nociception but also the anxiety- and depressive-like behaviors accompanying chronic inflammatory pain.

Our results also revealed the antidepressant and anxiolytic actions produced by the intraperitoneal treatment with HRW in animals with inflammatory pain. Indeed, while the normalization of the increase in immobility time observed in the TST and FST of animals with inflammatory pain demonstrated the antidepressant effects of HRW, the inhibition of the lower number of entries into the open arms in the EPM and of shorter time of permanence in the central area of the OF test proved the anxiolytic effects of this treatment. In accordance with us, the antidepressant properties of H_2_ in mice with depressive-like behaviors linked with stress [29,51] as well as the attenuation of the anxiety-like behaviors induced by morphine-withdrawal were also demonstrated [52]. Moreover, since HRW failed to alter the number of entries into the closed arms in the EPM or the number of squares crossed in the OF tests, its anxiolytic-like actions cannot be related to a motor impairment of the animal. Then, and considering that depression and anxiety are two important affective disorders and that both increase pain perception in animals with chronic pain [53], the anxiolytic and antidepressant effects of HRW, together with its pain-relieving actions, might permit treating inflammatory pain in a more effective and integrated form.

Several works demonstrated the crucial function played by the Nrf2/HO-1-NQO1 pathway in the effects produced by H_2_ in different experimental conditions [24,32,47]. Here, we evaluated the relevance of this pathway in the analgesic actions of HRW in inflammatory pain. The blockage of the painkiller actions of HRW with the systemic and local injection of ML385, SnPP, and dicoumarol revealed the significant contribution of the central and peripheral antioxidant system in the painkilling effects of HRW during inflammatory pain. These outcomes were corroborated by other studies performed with H_2_ [54] and with other compounds such as hydrogen sulfide donors and several antioxidants, which also activated the Nrf2/HO-1 and/or NQO1 pathway for alleviating chronic inflammatory, osteoarthritis, and neuropathic pain in rodents [5,21,45,55].

The effects of the intraperitoneal administration of HRW in the inflammatory, apoptotic, and oxidative alterations provoked by CFA in the paw and amygdala of animals with inflammatory pain were also assessed. Our data revealed the inhibitory effects induced by this treatment in the oxidative stress (4-HNE), inflammatory (p-IKBα), and apoptotic (BAX) reactions provoked by CFA by normalizing the up-regulation of these proteins in both tissues. The antioxidant properties of HRW treatment in the paw, together with the reversion of its antinociceptive actions with specific inhibitors of antioxidant enzymes, indicated that the endogenous antioxidant system is critical for the analgesic properties of HRW during inflammatory pain. Moreover, considering that treatment with NF-kB inhibitors decreased osteoarthritis [56] and inflammatory pain [57], the reversion of CFA-generated p-IKBα up-regulation in the paw with HRW treatment suggested that these anti-inflammatory actions might also be involved in its analgesic effects. Accordingly, other treatments, for instance, salvianolic Acid B, also ameliorated rheumatoid arthritis by downregulating the p-IKBα levels in joint tissues [58].

Finally, it is well known that the inflammatory changes and oxidative stress provoked by chronic pain in CNS have been considered the main neurobiological mechanisms involved in the development of mood disorders [48,59]. In compliance, the inhibition of the overexpression of 4-HNE and p-IKBα induced by HRW treatment in the amygdala, a crucial brain area implicated in the regulation of emotional responses [2], proved the antioxidant and anti-inflammatory actions of this treatment in the CNS and further suggested that these properties might be, in part, implicated in the anxiolytic and/or antidepressant effects of HRW during chronic inflammatory pain. This study further proved the anti-apoptotic activities induced by the systemic administration of HRW in the amygdala of animals with persistent chronic inflammatory pain as occurs in the paw, revealing the anti-apoptotic properties of H_2_ during chronic inflammatory pain as displayed in other health disorders [47]. Similarly, the protective role played by other gases, such as carbon monoxide, in modulating the apoptotic reactions caused by peripheral inflammation in the CNS has also been shown [60].

This study has some limitations, such as: (1) only male mice were used and these findings may be different in female mice; (2) the evaluation of the effects of HRW on the allodynia, hyperalgesia, depressive-, and anxiety-like behaviors induced by CFA was only performed at 1 h after HRW administration, thus precluding any conclusion about the possible long duration of these effects.

## 5. Conclusions

In summary, this study shows (1) that treatment with HRW inhibits the allodynia and hyperalgesia provoked by CFA and potentially avoids the emotional disorders related with chronic inflammatory pain, (2) the greater analgesic effectiveness of local versus the systemic administration of HRW, (3) the participation of the central and peripheral endogenous Nrf2/HO-1-NQO1 pathway in the analgesic actions of HRW, and (4) the positive effects of HRW treatment in the oxidative stress, apoptosis, and pro-inflammatory responses provoked by CFA in the paws and amygdala of animals with inflammatory pain. Thus, this research reveals new properties of H_2_ and proposes the usage of HRW as a potential therapy for persistent inflammatory pain and associated comorbidities.

## Data Availability

All of the data is contained within the article.

## References

[1] Binder A., Baron R. (2016). The Phar macological Therapy of Chronic Neuropathic Pain. Dtsch. Arztebl. Int..

[2] Narita M., Kaneko C., Miyoshi K., Nagumo Y., Kuzumaki N., Nakajima M., Nanjo K., Matsuzawa K., Yamazaki M., Suzuki T. (2006). Chronic pain induces anxiety with concomitant changes in opioidergic function in the amygdala. Neuropsychopharmacology.

[3] Hsu Y.C., Ma K.H., Guo S.L., Lin B.F., Tsai C.S., Yeh C.C. (2021). The occurrence of pain- induced depression is different between rat models of inflammatory and neuropathic pain. J. Clin. Med..

[4] Nicholson B., Verma S. (2004). Comorbidities in chronic neuropathic pain. Pain Med..

[5] Bai X., Batallé G., Pol O. (2021). The anxiolytic and antidepressant effects of diallyl disulfide and gyy4137 in animals with chronic neuropathic pain. Antioxidants.

[6] Kremer M., Becker L.J., Barrot M., Yalcin I. (2021). How to study anxiety and depression in rodent models of chronic pain?. Eur. J. Neurosci..

[7] Zhang X.H., Feng C.C., Pei L.J., Zhang Y.N., Chen L., Wei X.Q., Zhou J., Yong Y., Wang K. (2021). Electroacupuncture Attenuates Neuropathic Pain and Comorbid Negative Behavior: The Involvement of the Dopamine System in the Amygdala. Front. Neurosci..

[8] Fang A., Li Y., Wu X., Wu B., Zhang Y. (2020). Baicalin attenuates inflammatory pain associated depressive symptoms via Akt-mediated adult hippocampal neurogenesis. Metab. Brain Dis..

[9] Huang H.Y., Liao H.Y., Lin Y.W. (2020). Effects and Mechanisms of Electroacupuncture on Chronic Inflammatory Pain and Depression Comorbidity in Mice. Evid.-Based Complement. Alternat. Med..

[10] Chen J., Song Y., Yang J., Zhang Y., Zhao P., Zhu X.J., Su H.C. (2013). The contribution of TNF-α in the amygdala to anxiety in mice with persistent inflammatory pain. Neurosci. Lett..

[11] Parent A.J., Beaudet N., Beaudry H., Bergeron J., Bérubé P., Drolet G., Sarret P., Gendron L. (2012). Increased anxiety-like behaviors in rats experiencing chronic inflammatory pain. Behav. Brain Res..

[12] Guan S.Y., Zhang K., Wang X.S., Yang L., Feng B., Tian D.D., Gao M.R., Liu S.B., Liu A., Zhao M.G. (2020). Anxiolytic effects of polydatin through the blockade of neuroinflammation in a chronic pain mouse model. Mol. Pain.

[13] Miller R.E., Miller R.J., Malfait A.M. (2014). Osteoarthritis joint pain: The cytokine connection. Cytokine.

[14] Sebba A. (2021). Pain: A Review of Interleukin-6 and Its Roles in the Pain of Rheumatoid Arthritis. Open Access Rheumatol. Res. Rev..

[15] Yang Y., Sheng Q., Nie Z., Liu L., Zhang W., Chen G., Ye F., Shi L., Lv Z., Xie J. (2021). Daphnetin inhibits spinal glial activation via Nrf2/HO-1/NF-κB signaling pathway and attenuates CFA-induced inflammatory pain. Int. Immunopharmacol..

[16] Redondo A., Riego G., Pol O. (2020). The Antinociceptive, Antioxidant and Anti-Inflammatory Effects of 5-Fluoro-2-Oxindole during Inflammatory Pain. Antioxidants.

[17] Liu T., Zhang L., Joo D., Sun S.C. (2017). NF-κB signaling in inflammation. Signal Transduct. Target. Ther..

[18] Semmler A., Okulla T., Sastre M., Dumitrescu-Ozimek L., Heneka M.T. (2005). Systemic inflammation induces apoptosis with variable vulnerability of different brain regions. J. Chem. Neuroanat..

[19] Bhatt S., Nagappa A.N., Patil C.R. (2020). Role of oxidative stress in depression. Drug Discov. Today.

[20] Hashimoto K. (2018). Essential Role of Keap1-Nrf2 Signaling in Mood Disorders: Overview and Future Perspective. Front. Pharmacol..

[21] Ferreira-Chamorro P., Redondo A., Riego G., Leánez S., Pol O. (2018). Sulforaphane inhibited the nociceptive responses, anxiety-And depressive-like behaviors associated with neuropathic pain and improved the anti-allodynic effects of morphine in mice. Front. Pharmacol..

[22] Díaz A.F., Polo S., Gallardo N., Leánez S., Pol O. (2019). Analgesic and antidepressant effects of oltipraz on neuropathic pain in mice by modulating microglial activation. J. Clin. Med..

[23] Yao W., Zhang J.C., Ishima T., Ren Q., Yang C., Dong C., Ma M., Saito A., Honda T., Hashimoto K. (2016). Antidepressant effects of TBE-31 and MCE-1, the novel Nrf2 activators, in an inflammation model of depression. Eur. J. Pharmacol..

[24] Chen W., Zhang H.T., Qin S.C. (2021). Neuroprotective Effects of Molecular Hydrogen: A Critical Review. Neurosci. Bull..

[25] Fang W., Tang L., Wang G., Lin J., Liao W., Pan W., Xu J. (2020). Molecular Hydrogen Protects Human Melanocytes from Oxidative Stress by Activating Nrf2 Signaling. J. Investig. Dermatol..

[26] Ross D., Siegel D. (2021). The diverse functionality of NQO1 and its roles in redox control. Redox Biol..

[27] Zhao C., Yu S., Li J., Xu W., Ge R. (2017). Changes in IL-4 and IL-13 expression in allergic-rhinitis treated with hydrogen-rich saline in guinea-pig model. Allergol. Immunopathol..

[28] Fang S., Li X., Wei X., Zhang Y., Ma Z., Wei Y., Wang W. (2018). Beneficial effects of hydrogen gas inhalation on a murine model of allergic rhinitis. Exp. Ther. Med..

[29] Zhang Y., Su W.J., Chen Y., Wu T.Y., Gong H., Shen X.L., Wang Y.X., Sun X.J., Jiang C.L. (2016). Effects of hydrogen-rich water on depressive-like behavior in mice. Sci. Rep..

[30] Mizuno K., Sasaki A.T., Ebisu K., Tajima K., Kajimoto O., Nojima J., Kuratsune H., Hori H., Watanabe Y. (2018). Hydrogen-rich water for improvements of mood, anxiety, and autonomic nerve function in daily life. Med. Gas Res..

[31] Ge Y., Wu F., Sun X., Xiang Z., Yang L., Huang S., Lu Z., Sun Y., Yu W.F. (2014). Intrathecal infusion of hydrogen-rich normal saline attenuates neuropathic pain via inhibition of activation of spinal astrocytes and microglia in rats. PLoS ONE..

[32] Kawaguchi M., Satoh Y., Otsubo Y., Kazama T. (2014). Molecular hydrogen attenuates neuropathic pain in mice. PLoS ONE.

[33] Muley M.M., Krustev E., Mcdougall J.J. (2016). Preclinical Assessment of Inflammatory Pain. CNS Neurosci. Ther..

[34] Redondo A., Chamorro P.A.F., Riego G., Leánez S., Pol O. (2017). Treatment with sulforaphane produces antinociception and improves morphine effects during inflammatory pain in mice. J. Pharmacol. Exp. Ther..

[35] Steru L., Chermat R., Thierry B., Simon P. (1985). The tail suspension test: A new method for screening antidepressants in mice. Psychopharmacology.

[36] Porsolt R.D., Le Pichon M., Jalfre M. (1977). Depression: A new animal model sensitive to antidepressant treatments. Nature.

[37] La Porta C., Lara-Mayorga I.M., Negrete R., Maldonado R. (2016). Effects of pregabalin on the nociceptive, emotional and cognitive manifestations of neuropathic pain in mice. Eur. J. Pain..

[38] Carcolé M., Zamanillo D., Merlos M., Fernández-Pastor B., Cabañero D., Maldonado R. (2019). Blockade of the Sigma-1 Receptor Relieves Cognitive and Emotional Impairments Associated to Chronic Osteoarthritis Pain. Front. Pharmacol..

[39] Kraeuter A.K., Guest P.C., Sarnyai Z., Guest P. (2018). The Elevated Plus Maze Test for Measuring Anxiety-like Behavior in Rodents. Pre-Clinical Models.

[40] Kraeuter A.K., Guest P.C., Sarnyai Z., Guest P. (2018). The Open Field Test for Measuring Locomotor Activity and Anxiety-like Behavior. Pre-Clinical Models.

[41] Martínez-Serrat M., Martínez-Martel I., Coral-Pérez S., Bai X., Batallé G., Pol O. (2022). Hydrogen-Rich Water as a Novel Therapeutic Strategy for the Affective Disorders Linked with Chronic Neuropathic Pain in Mice. Antioxidants.

[42] Singh A., Venkannagari S., Oh K.H., Zhang Y.Q., Rohde J.M., Liu L., Nimmagadda S., Sudini K., Brimacombe K.R., Gajghate S. (2016). Small Molecule Inhibitor of NRF2 Selectively Intervenes Therapeutic Resistance in KEAP1-Deficient NSCLC Tumors. ACS Chem. Biol..

[43] Tseng C.K., Lin C.K., Wu Y.H., Chen Y.H., Chen W.C., Young K.C., Lee J.C. (2016). Human heme oxygenase 1 is a potential host cell factor against dengue virus replication. Sci. Rep..

[44] Cheng S.T., Hu J.L., Ren J.H., Yu H.B., Zhong S., Wai Wong V.K., Kwan Law B.Y., Chen W.X., Xu H.M., Zhang Z.Z. (2021). Dicoumarol, an NQO1 inhibitor, blocks cccDNA transcription by promoting degradation of HBx. J. Hepatol..

[45] Porta A., Rodríguez L., Bai X., Batallé G., Roch G., Pouso-Vázquez E., Balboni G., Pol O. (2021). Hydrogen sulfide inhibits inflammatory pain and enhances the analgesic properties of delta opioid receptors. Antioxidants.

[46] Iketani M., Ohsawa I. (2017). Molecular Hydrogen as a Neuroprotective Agent. Curr. Neuropharmacol..

[47] Tian Y., Zhang Y., Wang Y., Chen Y., Fan W., Zhou J., Qiao J., Wei Y. (2021). Hydrogen, a Novel Therapeutic Molecule, Regulates Oxidative Stress, Inflammation, and Apoptosis. Front. Physiol..

[48] Sheng J., Liu S., Wang Y., Cui R., Zhang X. (2017). The Link between Depression and Chronic Pain: Neural Mechanisms in the Brain. Neural Plast..

[49] Cunha A.M., Pereira-Mendes J., Almeida A., Guimarães M.R., Leite-Almeida H. (2020). Chronic pain impact on rodents’ behavioral repertoire. Neurosci. Biobehav. Rev..

[50] Roch G., Batallé G., Bai X., Pouso-Vázquez E., Rodríguez L., Pol O. (2022). The Beneficial Effects of Heme Oxygenase 1 and Hydrogen Sulfide Activation in the Management of Neuropathic Pain, Anxiety- and Depressive-like Effects of Paclitaxel in Mice. Antioxidants.

[51] Gao Q., Song H., Wang X.T., Liang Y., Xi Y.J., Gao Y., Guo Q.J., LeBaron T., Luo Y.X., Li S.C. (2017). Molecular hydrogen increases resilience to stress in mice. Sci. Rep..

[52] Wen D., Zhao P., Hui R., Wang J., Shen Q., Gong M., Guo H., Cong B., Ma C. (2017). Hydrogen-rich saline attenuates anxiety-like behaviors in morphine-withdrawn mice. Neuropharmacology.

[53] Lillywhite A., Woodhams S.G., Goncalves S.V., Watson D.J.G., Li L., Burston J.J., Gowler P.R.W., Canals M., Walsh D.A., Hathway G.J. (2021). Anxiety enhances pain in a model of osteoarthritis and is associated with altered endogenous opioid function and reduced opioid analgesia. Pain Rep..

[54] Li Y., Shen C., Zhou X., Zhang J., Lai X., Zhang Y. (2022). Local Treatment of Hydrogen-Rich Saline Promotes Wound Healing in Vivo by Inhibiting Oxidative Stress via Nrf-2/HO-1 Pathway. Oxidative Med. Cell. Longev..

[55] Batallé G., Bai X., Pouso-Vázquez E., Roch G., Rodríguez L., Pol O. (2021). The recovery of cognitive and affective deficiencies linked with chronic osteoarthritis pain and implicated pathways by slow-releasing hydrogen sulfide treatment. Antioxidants.

[56] Imagawa K., de Andrés M.C., Hashimoto K., Pitt D., Itoi E., Goldring M.B., Roach H.I., Oreffo R.O. (2011). The epigenetic effect of glucosamine and a nuclear factor-kappa B (NF-kB) inhibitor on primary human chondrocytes--implications for osteoarthritis. Biochem. Biophys. Res. Commun..

[57] Lee K.M., Kang B.S., Lee H.L., Son S.J., Hwang S.H., Kim D.S., Park J.S., Cho H.J. (2004). Spinal NF-kB activation induces COX-2 upregulation and contributes to inflammatory pain hypersensitivity. Eur. J. Neurosci..

[58] Xia Z.B., Yuan Y.J., Zhang Q.H., Li H., Dai J.L., Min J.K. (2018). Salvianolic Acid B Suppresses Inflammatory Mediator Levels by Downregulating NF-κB in a Rat Model of Rheumatoid Arthritis. Med. Sci. Monit..

[59] Liao D., Lv C., Cao L., Yao D., Wu Y., Long M., Liu N., Jiang P. (2020). Curcumin Attenuates Chronic Unpredictable Mild Stress-Induced Depressive-Like Behaviors via Restoring Changes in Oxidative Stress and the Activation of Nrf2 Signaling Pathway in Rats. Oxidative Med. Cell. Longev..

[60] Cazuza R.A., Batallé G., Bai X., Leite-Panissi C.R.A., Pol O. (2022). Effects of treatment with a carbon monoxide donor and an activator of heme oxygenase 1 on the nociceptive, apoptotic and/or oxidative alterations induced by persistent inflammatory pain in the central nervous system of mice. Brain Res. Bull..

